# A Case of Successful Rehabilitation for Controlling Chronic Pain Following Osteonecrosis of the Femoral Head in a Young Adult Cancer Survivor

**DOI:** 10.7759/cureus.52120

**Published:** 2024-01-11

**Authors:** Tomoki Aoyama, Mari Matsuoka

**Affiliations:** 1 Department of Physical Therapy, Human Health Sciences, Graduate School of Medicine, Kyoto University, Kyoto, JPN; 2 Department of Child Health Nursing, Mie University Graduate School of Medicine, Tsu city, JPN

**Keywords:** self-efficacy, physical medicine and rehabilitation, mindset, chronic pain, osteonecrosis of the femoral head, cancer survivor, adolescent and young adult

## Abstract

A 26-year-old woman previously treated for acute lymphoblastic leukemia (ALL) 14 years ago faced challenges in managing chronic pain resulting from right femoral head necrosis, a complication of her earlier ALL treatment. Ultimately, the persistent chronic pain was successfully treated via a comprehensive rehabilitation approach. The patient presented with hip contractures, muscle weakness, and reduced endurance without evident arthropathic changes or inflammatory findings in the femoral head. Active physiotherapy was implemented with the primary objective of increasing her social activity. This therapeutic intervention effectively managed the severe pain without the necessity for analgesic drugs, leading to a significant improvement in the patient's social activity. Recognizing the adolescent and young adult age group as a critical phase of physical, psychological, and social development, cancer survivors within this age group require multimodal care. This study highlights the role of stepwise rehabilitation treatments involving stretching, muscle strengthening, and endurance training, particularly in challenging cases of chronic pain. Post-treatment interviews revealed that successful experiences in each movement contributed to increased self-efficacy and promoted not only the control of chronic pain but also fostered improvements in social activities.

## Introduction

The adolescent and young adult (AYA) age group refers to individuals aged 15-39 years. During this time, individuals undergo significant physical, psychological, and social growth [1]. AYA cancer survivors who underwent cancer treatment during childhood have different concerns and needs from those of other family members and health care professionals. Consequently, these individuals require careful attention from both healthcare providers and family members to address their specific challenges [1,2]. Beyond physical pain, psychosocial factors play a major role in childhood pain experiences. Therefore, a comprehensive assessment is essential to assess the extent to which physical and psychosocial factors are involved before addressing the physical and mental aspects of pain [3].

Adult idiopathic femoral head osteonecrosis poses a considerable challenge in terms of treatment [4]. It is closely associated with high steroid doses and occurs in 33.1% of patients with systemic lupus erythematosus and 3.6% of patients undergoing renal transplantation [5]. Although high-dose steroids are used in the treatment of childhood acute lymphoblastic leukemia (ALL), femoral head osteonecrosis occurs in only 0.8% of patients with childhood ALL [6]. Osteonecrosis secondary to childhood-onset ALL has a favorable prognosis, as conservative treatment often leads to necrotic bone remodeling [6]. The prognosis for adult osteonecrosis of the femoral head varies depending on the location and extent of necrosis in the bone head; once the area of necrosis is established, it remains unchanged [7]. Effective pain control is typically achieved if arthropathic changes do not progress.

This report presents a patient with a history of childhood acute lymphoblastic leukemia (ALL) in whom rehabilitation medicine proved successful in managing chronic pain resulting from osteonecrosis of the right femoral head, a complication arising from ALL treatment.

## Case presentation

A 26-year-old woman with a history of childhood ALL was referred to the Department of Rehabilitation due to severe right hip pain. The patient was initially diagnosed with ALL at the age of 12 and underwent chemotherapy with prednisolone and dexamethasone treatments (Japan Children's Leukemia Study Group ALL02 protocol). At the age of 13, she reported right hip pain and back pain, leading to a magnetic resonance imaging (MRI) examination, which did not reveal specific lesions. Chemotherapy was continued, and at the age of 14, the patient's ALL went into remission. However, one year later, the patient complained of slight right hip pain, prompting a radiograph that revealed flattening of the right femoral head, diagnosed as necrosis. Conservative treatment was continued. Several years later, at the age of 26, the patient's pain was challenging to manage despite the concomitant use of tramadol hydrochloride (25 mg), which resulted in severe nausea, and loxoprofen (60 mg), resulting in a pain score of five on a numerical rating scale (NRS) (Table 1). At the time of the examination, she was living with her parents and sister. She exhibited a reduced range of motion and muscle weakness in the right hip (Table 1). Specifically, the patient's right hip abduction was limited to 10°, and she complained of severe pain when the range of motion of abduction was measured. A gait evaluation revealed that the trunk was rotated to the right during the mid-to-late phase of the right stance leg. The patient was able to perform all basic daily activities independently, and the Bathel index score was perfect. However, she had difficulty walking long distances.

**Table 1:** Physiological assessment NRS - numerical rating scale

Assessment		Initial examination		At the end of treatment (5 months later)		At the last follow-up (2 years later)	
NRS		5		3		1	
Range of motion (degree)		Right	Left	Right	Left	Right	Left
Hip	Flexion	90	110	100	110	110	110
	Extension	5	10	5	10	5	10
	Abduction	10	30	15	30	20	50
	Outer rotation	30	30	35	35	35	30
	Inner rotation	5	5	10	10	10	10
Knee	Extension	5	5	5	5	5	5
Manual muscle test		Right	Left	Right	Left	Right	Left
Hip	Flexion	3	4	4	4	5	5
	Extension	3	4	4	5	5	5
	Abduction	3	4	4	4	4	4
Knee	Flexion	5	5	5	5	5	5
	Extension	3	4	4	4	5	5
Ankle	Abduction	3	4	4	5	4	5

**Figure 1:**
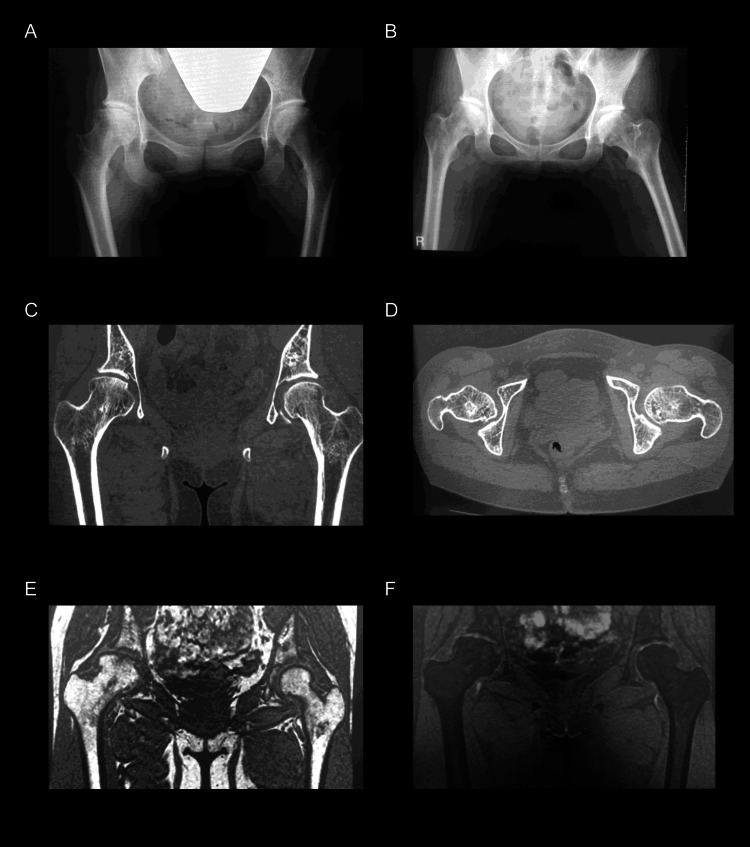
Imaging evaluation findings A) Frontal radiography of the hip joint; B) Frontal radiography of the hip joint during abduction. No impingement of the acetabulum is observed during abduction of the right femoral head; C) Coronal computed tomography of the hip joint; D) Transverse computed tomography of the hip joint is shown. Bone sclerosis is observed in the right femoral head, and the necrotic bone is remodeled; E) Coronal T1-weighted magnetic resonance imaging; F) Coronal fat-suppressed magnetic resonance imaging reveals no signs of inflammation.

All the images were taken at the time the Department of Rehabilitation was consulted (Figure 1). A frontal radiograph revealed mild flattening of the right femoral head, though no arthropathic changes were observed (Figure 1A). The flattened head did not impinge on the acetabulum, indicating that the limitation of range of motion was not bony in origin but soft tissue-induced, mainly due to contractures (Figure 1B). Computed tomography (CT) revealed that the necrotic area of the head was remodeled (Figures 1C and 1D), and no inflammatory findings were observed on magnetic resonance imaging (Figures 1E and 1F).

**Figure 2:**
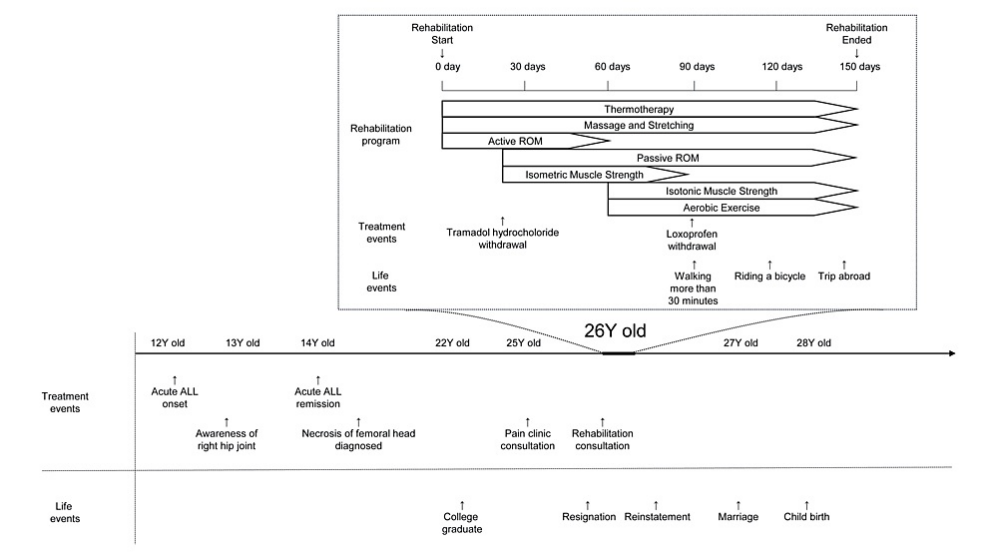
Medical history and rehabilitation program ROM - range of motion; ALL - acute lymphoblastic leukemia

This lack of inflammatory signs suggested a low probability of collapsing and a reduced risk of exacerbation of inflammatory symptoms. Based on the image analysis, we considered the possibility of aggravation of osteonecrosis of the femoral head due to exercise therapy to be low. Consequently, an aggressive exercise therapy was formulated. First, physical therapy, such as thermotherapy, was started to alleviate pain and improve the contractured joint, and stretching was performed. As the contractures improved, exercise therapy centering on joint range-of-motion training and muscle-strengthening training was implemented step by step, and endurance-strengthening training using an ergometer was also performed (Figure 2).

After 20 days of rehabilitation treatment, the tramadol hydrochloride was discontinued at the patient's request due to pain relief. After 90 days of treatment, the loxoprofen was discontinued, and the patient's right hip passive range of motion and muscle strength had improved. She was more active during exercise. After 150 days of treatment, the patient's pain NRS decreased to three, and the range of motion and muscle strength in her right hip improved without medication (Table 1). Although the gait abnormality persisted, the patient showed marked improvement and was able to ride a bicycle and travel (Figure 2). At the completion of the rehabilitation treatment, the patient continued self-stretching and strengthening exercises. At a two-year follow-up visit, the patient's pain NRS was one without the need for anti-inflammatory analgesic medication, and her hip function had further improved (Table 1). The patient's activity level significantly improved, and she has returned to work, married, had a baby, and is leading an active social life (Figure 2). Changes in the patient’s mindset before and after rehabilitation treatment were investigated via interview (Table 2).

**Table 2:** Changes in feelings before and after rehabilitation treatment

Category	Before treatment	After treatment
Pain	Pain that threatens my life	Pain-free
	I always feared for uncontrolled pain	
Efforts for pain relief	I gave up on pain relief efforts	I'm pleased there are efforts I can do about my pain
	I didn't know what I can do	I understand the cause and coping strategies of my pain
		I can move my leg with confidence
Relationship with medical professionals	I didn't feel the commitment of the medical staff	I feel the commitment of the medical staff
		I receive professional support
Self-motivation (mindset)	There were more and more things I couldn't do.	I feel the effects of rehabilitation
	I convinced my self that I couldn't do anything due to the pain	I can engage in rehabilitation on my own positively
		I feel confident that I can do it by myself
		I get confidence in my own body
		I'm willing to try more things

## Discussion

In this case, rehabilitation medicine demonstrated notable success in effectively managing chronic pain that had proven challenging to control. Initial imaging assessment revealed no inflammation or arthropathic changes in the hip joint, which led us to believe that the pain and functional decline was due to joint contractures. This understanding prompted a gradual increase in exercise intensity, and the patient's own awareness of the improvement in function fostered increased confidence, increased activity levels, and a heightened ability to self-regulate pain.

Chronic pain is defined as pain persisting beyond the normal tissue healing time, which is typically three months in the absence of other factors [8]. Chronic pain can be divided into nociceptive, neuropathic, and non-physiological pain based on its pathogenesis, though it is difficult to distinguish between the types of chronic pain [8]. Patients with chronic pain overestimate the disability caused by present and future pain, leading to catastrophic thinking and complicating pain resolution [9]. Psychosocial factors influencing pain, especially in childhood, include individual factors such as sensory sensitivity, depression, low self-efficacy, and poor stress-coping behaviors, as well as environmental factors such as inadequate medical care, stress, and difficulties in adapting to school and sports activities [3]. The treatment objective should extend beyond merely eliminating pain to embrace a multidisciplinary approach. This approach involves empowering the patient to better manage pain, mitigating stressors through environmental adjustments, and enhancing self-efficacy [3]. In this report, the patient's self-efficacy improved, and her mindset changed (Table 2). The patient reported, "I feel confident that I can do it by myself," and that "I'm willing to try more things". She also reported improved environmental factors, such as, "I feel the commitment of the medical staff" and "I receive professional support". These factors may have contributed to the resolution of the complex factors that constitute chronic pain, pain control, and improved quality of life after the withdrawal of analgesics. Through the rehabilitation process, in this case, as exercise therapy methods and intensity progressively increased, the patient herself experienced and achieved a sense of accomplishment through the actual relief of pain, coupled with encouragement from the physical therapist. We consider that this exercise therapy process may have increased her self-efficacy, aligning with Bandura's self-efficacy model components, namely "verbal persuasion," "performance accomplishments," and physiological and affective states" [10].

Patients in the AYA age group experience significant physical, psychological, and social growth [1]. Understanding their concerns and needs can be challenging [11,12]. This report highlights the need to establish a long-term follow-up system aimed at maintaining and improving the quality of life of individuals aged 15-39 years through the provision of multimodal care [1,3].

## Conclusions

This case report illustrates a successful rehabilitation outcome in managing chronic pain following osteonecrosis of the right femoral head, a complication arising from childhood ALL treatment. The gradual implementation of physical therapy may have contributed to the patient's sense of accomplishment and increased self-efficacy, resulting in successful self-management of pain.
